# Nucleotide based covalent inhibitors of KRas can only be efficient *in vivo* if they bind reversibly with GTP-like affinity

**DOI:** 10.1038/s41598-017-03973-6

**Published:** 2017-06-16

**Authors:** Matthias P. Müller, Sadasivam Jeganathan, Angelika Heidrich, Jeremy Campos, Roger S. Goody

**Affiliations:** 1 Department of Structural Biochemistry, Max Planck Institute of Molecular Physiology, Otto-Hahn-Straße 11, 44227 Dortmund, Germany; 2 Faculty of Chemistry and Chemical Biology, Dortmund University of Technology, Otto-Hahn-Straße 4a, 44227 Dortmund, Germany

## Abstract

Simple reversible competitive inhibition of nucleotide binding of GTP to Ras family GTPases has long been recognized as an unlikely approach to manipulating the activity of such proteins for experimental or therapeutic purposes. This is due to the high affinity of GTP to GTPases coupled with high cellular GTP concentrations, but also to problems of specificity for the highly conserved binding sites in GTPases. A recent approach suggested that these problems might be overcome by using GDP derivatives that can undergo a covalent reaction with disease specific mutants, in particular addressing inhibition of KRas_G12C_ using GDP equipped with an electrophilic group at the β-phosphate. We show here that a major drawback to this approach is a loss of reversible affinity of such β-modified derivatives for Ras of at least 10^4^ compared to GTP and GDP. With the help of a thorough kinetic characterization, we show that this leads to covalent reaction times that are too slow to make the compounds attractive for intracellular use, but that generation of a hypothetical reactive GDP derivative that retains the high reversible affinity of GDP/GTP to Ras might be a viable alternative.

## Introduction

It has been known for many years that mutations in Ras proteins, particularly KRas, are important causative factors in many human cancers^1^. Consequently, there have been many attempts to generate strategies to inhibit the activity of Ras, and if possible specifically mutated Ras, in tumors^2^. Strategies involving direct competition with GTP binding in analogy to kinase inhibitors that compete with ATP binding were not considered seriously for many years, since it has been apparent for over 25 years that GTP and GDP bind with extremely high affinity to Ras proteins^3, 4^, meaning that inhibitors would have to compete with several hundred µM concentrations of GTP/GDP binding with sub-nM affinity to their targets. Alternative strategies that were pursued include inhibition of post-translational farnesylation^5^, an approach that has led to the development of highly potent inhibitors of farnesyl transferase. However, the promise of these compounds has so far not been fulfilled at the clinical stage, partly because other enzymes, in particular geranylgeranyl transferase 1, can, at least partially, replace the activity of farnesyl transferase^6^. A further approach is less direct and is aimed at the prenyl-binding protein PDEδ, which plays an important role in the regulation of Ras localization at the plasma membrane. Selective inhibitors of this factor offer a possible approach to modulation of Ras activity^7^.

There has recently been renewed interest in directly targeting the Ras molecule^8^, and one example of this is the work of the Shokat laboratory using small molecules that react covalently with one of the most commonly found mutants in Ras dependent lung cancer, the G12C mutant of KRas^9^. Here, a specific mutation occurring with high frequency in lung tumors, KRas_G12C_, was targeted using SH-specific reagents. One of the problems of the approach outlined is that a high degree of specificity for a particular cysteine residue in a specific protein will only be achievable if there is some mechanism of steering the compound to this site. A more recent extension of this work suggests that there is indeed a certain specificity of the reagents developed^10^, but it is not clear whether this will be sufficient for the envisaged applications.

A classical manner of achieving selectivity is to exploit the specificity and affinity of a protein for its substrate, product or other natural ligand and to modify these with groups capable of undergoing a covalent interaction with the protein. At the same time as the report of the Shokat group, a publication applying this approach utilized GDP molecules modified on the ß-phosphate with a reactive group^11, 12^. These derivatives were shown to be able to bind to and interact covalently with the cysteine group of the G12C mutant of KRas. However, while this is an encouraging result, there is a good reason to believe that the substances described will not be effective in cells, even if the problem of intracellular delivery of the nucleotide analogs is satisfactorily solved, a point that is also addressed in the initial publication. The main cause for concern is that modification of the ß-phosphate is likely to lead to a substantial loss of reversible affinity to GTPases, thus reducing the “affinity advantage” of such substances. With the exception of a very recent publication by Xiong *et al*.^12^ there was very little data on the effect of such modifications on GDP affinity for GTPases in the literature, so we decided to assess this using a model derivative, the β-methyl ester of GDP (GDPβMe). We show that the addition of the methyl group to the β-phosphate does indeed lead to a dramatic loss of affinity to KRas. We then proceeded to assess the properties of the covalent inhibitor SML-8-73-1^11^ in terms of its kinetics of interaction with KRas and its rate of reaction with KRas_G12C_. Using this data, we simulated and compared the likely effect of a β-phosphate modified covalent inhibitor with that of a putative covalent inhibitor that retains the high affinity of GDP to Ras proteins in the cellular situation. Our data indicate that a nucleotide competitive inhibitor can only work if it retains the high reversible affinity of GDP/GTP, even if the inhibitor can react covalently with the Ras protein.

## Results and Discussion

### Determination of the affinity and kinetics of interaction of GDPβMe with KRas

**Figure 1:**
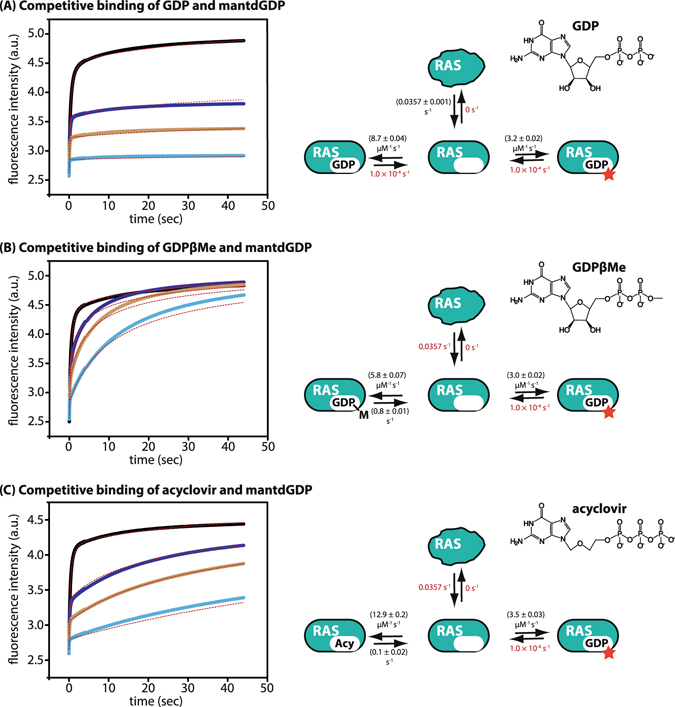
Interaction of KRas with different nucleotides. In order to quantify the interaction of nucleotide-free KRas, competitive binding experiments between mantdGDP and GDP (**A**), GDPβMe (**B**) and acyclovir triphosphate (**C**) were performed. In a stopped-flow apparatus, 1 µM KRas was shot against 1 µM mantdGDP in the absence (black curve) or in the presence of 0.5 µM (blue), 1 µM (orange) or 3 µM (turquoise) competing nucleotide and the binding curves were globally fit to the model on the right of the diagram to obtain the corresponding binding constants (indicated in black, previously known kinetic constants that were fixed during fitting are indicated in red). In all experiments, a second slow phase was observed that could only be explained by a model in which nucleotide-free KRas is in an equilibrium between a competent (70–80%) and a non-competent (20–30%) nucleotide-binding state, which might arise from partly unfolded protein due to the inherent instability of KRas in the absence of nucleotides.

The determination of affinities of nucleotides to Ras proteins is not a trivial exercise, which was originally the source of underestimates of the affinities of GTP and GDP^4^. A method which leads to estimates of the kinetic parameters as well as the affinity involves stopped-flow measurements of association of fluorescent nucleotides with nucleotide-free Ras followed by competiton between fluorescent and non-fluorescent nucleotides to obtain the parameters for the non-labelled substances^3^. As an example we show the competition between mantdGDP and GDP in Fig. 1A. Here, GDP competes with the binding of mantdGDP and this results in a lower signal amplitude, meaning that less mantdGDP is bound than in the absence of GDP. In the case of GDP competing with mantdGDP, the individual binding reactions can be considered to be essentially irreversible on the time scale of the experiments, and under the assumption that the ligands are in excess over the protein (pseudo first order conditions), it can be shown that the observed first order rate constant for the binding transient is given by

1 $${{\rm{k}}}_{{\rm{obs}}}={k}_{{\rm{GDP}}}\cdot [{\rm{GDP}}]+{{\rm{k}}}_{{\rm{mdGDP}}}\cdot [{\rm{mdGDP}}]$$

and the relative amplitude in the presence and absence of GDP is given by

2 $$\frac{{{\rm{\Delta }}{\rm{F}}}_{{\rm{GDP}}/{\rm{mdGDP}}}}{{{\rm{\Delta }}{\rm{F}}}_{{\rm{mdGDP}}}}=\frac{1}{1+\frac{{{\rm{k}}}_{{\rm{GDP}}}\cdot [{\rm{GDP}}]}{{{\rm{k}}}_{{\rm{mdGDP}}}\cdot [{\rm{mdGDP}}]}}$$

where ΔF_mdGDP_ is the amplitude of change in fluorescence intensity on mixing Ras and mdGDP, and ΔF_GDP/mdGDP_ is the amplitude in the presence of GDP and mdGDP.

Thus, according to equation 1), the observed rate constant for mantdGDP association with Ras will increase linearly with increasing GDP concentration, while the relative amplitude of the signal will decrease hyperbolically from 1 towards zero^3, 13^. At high concentrations of ligands, the dependence of the observed pseudo first order rate constant for the association reaction deviates from linearity and is in fact hyperbolic due to a 2-step association reaction^3^, but this is of no consequence for the present discussion. In the present work, we have not used pseudo first order conditions due to the easy availability of software that allows determination of rate constants without use of the more classical approach of considering one reagent to be in large excess over the other. The values obtained by global fitting of the data from the 4 experiments shown in Fig. 1A are 3.2 × 10^6^ M^−1^s^−1^ for *k*_*mdGDP*_ and 8.2 × 10^6^ M^−1^s^−1^ for *k*_*GDP*_. It should be noted that there is no information on the dissociation rate constants from these experiments, and they were fixed at the value reported for HRas (k_off_ = ca. 10^−4^ s^−1^)^3^.

As described earlier, a different situation arises if the nucleotide competing with mantdGDP is in very rapid (and therefore weak) equilibrium with its bound form^3^. Thus, in the presence of the weakly bound GMP (K_d_ = 29 µM) or guanosine (K_d_ = 153 µM), the observed rate constant decreases rather than increases as the concentration of competitor increases^3^, but the amplitude of the signal change stays constant because these weakly bound ligands cannot compete effectively in terms of the distribution between the bound species at equilibrium. In this situation, the weakly bound ligand is essentially buffering the concentration of free Ras and thus lowering its availability for the binding reaction.

A different type of behavior is seen for competing nucleotides that bind weakly, but are not in rapid equilibrium with their bound form, and this type of behavior was seen with ATP in earlier work^14^. In this case, the binding transients do not follow simple exponential behavior (or hyperbolic when similar concentrations of Ras and fluorescent nucleotide are used), and this is what we observe in the present work using GDPβMe in competition with mantdGDP, as shown in Fig. 1B. Mixing of 1 µM KRas with 1 µM mantdGDP shows the expected hyperbolic transient, but in the presence of increasing concentrations of GDPβMe, the transients become increasingly biphasic, with a rapid initial phase followed by a slow phase, but with the same overall amplitude. The constancy of the amplitude shows that at the highest concentration used (3x excess of GDPβMe over mantdGDP), there is no significant inhibition of mantdGDP binding at equilibrium. The behavior can be explained by effective competition of mantdGDP association by GDPβMe, followed by dissociation of initially bound GDPβMe and its replacement by mantdGDP. Based on this model, a global fit to the data of Fig. 1B leads to values of 5.8 × 10^6^ M^−1^s^−1^ for k_on_ and 0.8 s^−1^ for k_off_ for GDPβMe. Thus, GDPβMe is released approximately 10^4^ times faster than GDP, which has a k_off_ of ca. 10^−4^ s^−1^ (ref. 3). Since the association rate constants of GDP and GDPβMe are similar, this means that the affinity of KRas for GDPβMe is ca. 4 orders of magnitude lower than for GDP (or GTP, which is bound with similar affinity).

### Determination of the affinity and kinetics of interaction of SML-8-73-1 with KRas_WT_

**Figure 2:**
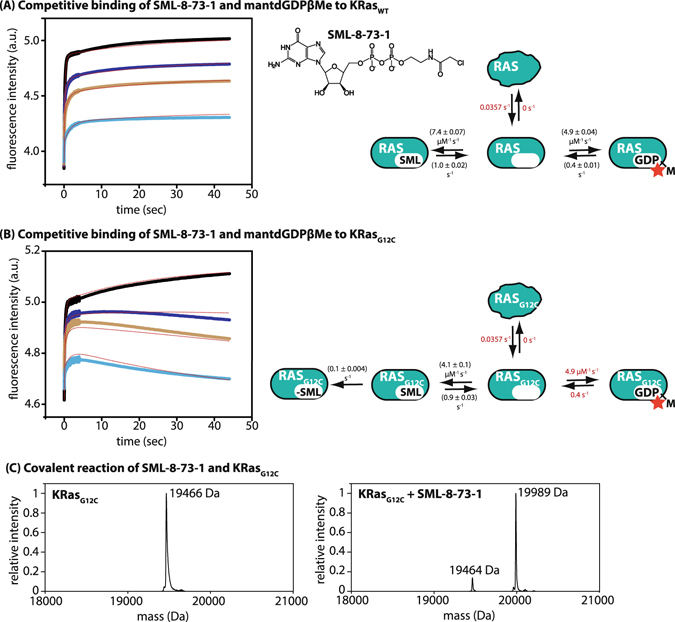
Reversible and irreversible interaction of the inhibitor SML-8-73-1 with KRas. (**A**) In order to quantify the reversible binding of SML-8-73-1, competition experiments of KRas_WT_ binding to mantdGDPβMe in the absence (black curve) or in the presence of 0.5 µM (blue), 1 µM (orange) or 3 µM (turquoise) SML-8-73-1 were performed and kinetic constants were obtained by global fitting of the data to the model on the right. (**B**) Irreversible binding was subsequently quantified by repeating the experiments from 2 A, but using KRas_G12C_ instead of the wild type protein. The additional phase observed in these experiments in the presence of SML-8-73-1 corresponding to displacement of initially bound mantdGDPβMe because of covalent reaction of the inhibitor allowed determination of the rate constant of covalent reaction of SML-8-73-1 with KRas_G12C_. (**C**) Covalent reaction was confirmed by mass spectrometry. The mass spectra of nucleotide-free KRas_G12C_ before (left) and after (right) addition of SML-8-73-1 and quenching with 5 mM DTE after 20 s are shown.

For some of the experiments described below, it was desirable to have a fluorescent derivative of GDPβMe. As in the case of GDP and GTP, a methylanthraniloyl group at the 3′ position of dGDP-β-methyl ester was suitable for this purpose, and the increase in fluorescence on binding to KRas could be used to obtain the kinetic parameters for the interaction (Fig. 2A). The association rate constant was found to be 4.9 × 10^6^ M^−1^ s^−1^, and the dissociation rate constant 0.4 s^−1^, suggesting only a minor effect of the fluorescent group on the interaction properties by comparison with the values for GDPβMe given above. These properties could be used in a competition experiment with SML-8-73-1 to allow determination of the kinetics of interaction of this derivative with wild type KRas, a situation in which covalent reaction does not occur. The association rate constant was 7.4 × 10^6^ M^−1^ s^−1^ and the off-rate constant was 1 s^−1^, similar to the values obtained for GDPβMe and confirming our suspicion of a dramatic weakening of the affinity to KRas by modification of the β-phosphate group.

### Determination of the affinity and kinetics of interaction of SML-8-73-1 with KRas_G12C_

Repeating the experiments shown in the previous section with KRas_G12C_, the covalent reaction could be observed in a slow phase after establishing the initial distribution of reversibly bound species (i.e. KRas_G12C_:mantGDPβMe and KRas_G12C_:SML-8-73-1) on mixing KRas_G12C_ with a mixture of mantdGDPβMe and SML-8-73-1 (Fig. 2B). This slow phase was due to displacement of the originally bound mantdGDPβMe because of irreversible covalent reaction of SML-8-73-1 with KRas_G12C_. The association and dissociation rate constants of SML-8-73-1 (k_on_ = 4.1 × 10^6^ M^−1^ s^−1^, k_off_ = 0.9 s^−1^) were similar to those seen for KRas_WT_, and the fitted rate constant for the covalent reaction was 0.1 s^−1^. After initial submission of the current contribution, a publication appeared reporting a more thorough characterization of SML-8-73-1 than in the earlier publications^12^. In this publication, a rate constant of 0.014 s^−1^ was reported for the covalent reaction, with a K_d_ value of 0.009 µM for the reversible binding step. Both of these values deviate significantly from those in our current work (k_inact_ = 0.1 s^−1^, K_d_ = 0.14 µM from Fig. 2A,B. While we have no explanation for these differences, the reversible binding affinity and the rate of covalent reaction reported in by Xiong *et al*.^12^ are incompatible with our data in Fig. 2A,B as well as with the mass spectrometric data reported in the last section of our results. We are therefore confident of the values for the kinetic constants for SML-8-73-1 binding to and covalently reacting with KRas_G12C_.

An independent confirmation of the rapid covalent reaction of SML-8-73-1 with KRas_G12C_ was obtained by mixing the compound with the nucleotide-free protein followed by ESI-MS. Here we saw that the rate of reaction was too rapid to monitor reliably by this method (Fig. 2C), but it could be seen that modification occurred over a period of tens of seconds to minutes, in keeping with the rate constant from Fig. 2B (*k*_*inact*_ = ca. 0.1 s^−1^, corresponding to a half-life of ca. 7 seconds). However, the slower rate constant reported by Xiong *et al*. (half-life of ca. 50 seconds) would be excluded by this result.

### Determination of the affinity and kinetics of interaction of acyclovir triphosphate with KRas

The results obtained with GDPβMe and SML-8-73-1 show that removal of a negative charge and introduction of the small methyl group or the larger group of SML-8-73-1 at the β-phosphate of GDP leads to a large effect on the affinity of KRas for the nucleotide, with the major effect being on the drastically increased dissociation rate constant. In contrast, there is only a minor effect on the affinity with the highly modified ribose residue in mantdGDP. Thus, the known interactions of Asp30 with the 2′- and 3′- hydroxyl groups of the ribose moiety^15^ do not appear to be essential for strong binding. The question then arises as to the importance of this part of the structure, and whether more drastic modifications might be tolerated, for example in the design of a covalent inhibitor that harbors unmodified phosphate residues. For this reason, we tested the interaction properties of acyclovir triphosphate with KRas. Acyclovir has a 2-hydroxyethoxymethyl instead of a ribose group attached to the N9 position of adenine. It can therefore be envisaged as an “open chain” version of guanosine, and in fact retains an ether oxygen atom at a position equivalent to the ring oxygen of ribose in adenosine that could potentially interact with Lys117 in Ras, as seen in Ras-nucleotide complex structures^15^. Interestingly, acyclovir triphosphate showed qualitatively similar behavior to that of GDPβMe in the competitive stopped-flow assay, suggesting efficient initial recognition, but rapid dissociation (Fig. 1C). The association rate constant (12.9 × 10^6^ M^−1^s^−1^) was actually slightly larger than for GDP (probably a result of the triphosphate as opposed to the diphosphate structure) and significantly greater than for GDPβMe, and the dissociation rate constant at 0.1 s^−1^ was a factor of 10 smaller than for GDPβMe, so that the K_d_ value at 8.5 nM was considerably lower than that of GDPβMe (130 nM), but still orders of magnitude higher than for GDP (20 pM).

### The influence of SOS on the rate of dissociation of β-modified GDP analogs from KRas

**Figure 3:**
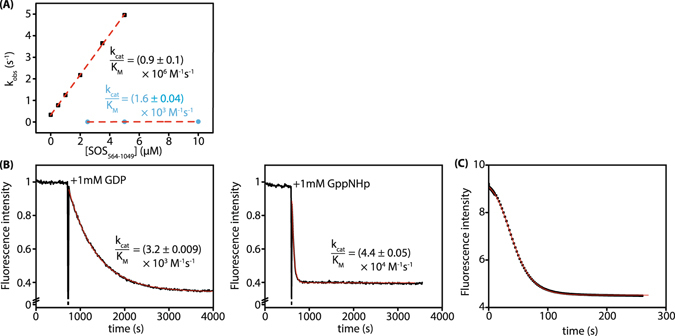
SOS catalyzed nucleotide exchange. (**A**) Comparison of SOS catalyzed nucleotide exchange on 0.2 µM KRas:mantdGDP (turquoise) or 0.2 µM KRas:mantdGDPβMe (black) in the presence of varying excess SOS concentrations. Linear fits yield *k*_*cat*_ */K*_*M*_ values of 1.6 × 10^3^ M^−1^s^−1^ and 0.9 × 10^6^ M^−1^ s^−1^, respectively. (**B**) In order to quantify the effects of SOS activation via the allosteric Ras binding site, 10 µM Ras:mdGDP/0.5 µM SOS_564–1049_ were preincubated in a cuvette and the reaction was started by addition of 1 mM GDP (left) or 1 mM GppNHp (right). The large difference in the velocity of the two exchange reactions is due to Ras binding to the allosteric site only in the active GppNHp-bound form. Because of the high velocity under the conditions chosen, the intial change in amplitude could not be observed upon exchange with GppNHp. (**C**) Reaction as in B repeated in a stopped-flow device. 10 µM Ras:mdGDP was shot against 1 µM SOS_564–1049_ and 1 mM GppNHp. The sigmoidal shape of the beginning of the curve arises from allosteric activation by the generated Ras:GppNHp.

In order to be able to predict the possible rate of inactivation of KRas_G12C_ in the cellular context by SML-8-73-1, it is important to understand the influence of exchange factors on the rate of dissociation from KRas. To provide evidence on this, we monitored the effect of the Ras specific exchange factor SOS on mantdGDPβMe release from its complex with KRas. As shown in Fig. 3, mixing SOS_564–1049_ in excess over KRas:mantdGDPβMe in the presence of excess GDP led to a linear increase in the rate constant for nucleotide dissociation up to at least 5 s^−1^, and the slope of the fitted straight line to the data gave a *k*_*cat*_ */K*_*M*_ value for this reaction of 0.9 × 10^6^ M^−1^ s^−1^. The maximal rate constant for nucleotide release is presumably much faster than 5 s^−1^. Interestingly, catalysis of mantdGDP release was much less efficient, with a *k*_*cat*_ */K*_*M*_ value of ca. 1.6 × 10^3^ M^−1^ s^−1^. It should be noted that the SOS construct used contained both the GEF domain (Cdc25 domain) and the activator domain that can bind Ras:GTP, leading to an activation of the GEF activity of the Cdc25 domain^16, 17^. Since Ras:GTP was not present in the experiments performed, we were essentially monitoring the “inhibited” activity of SOS towards KRas. The “uninhibited” activity is presumably reflected by that of an isolated Cdc25 domain, albeit from a different protein and with HRas rather than KRas, which has a *k*_*cat*_ */K*_*M*_ value of 4 × 10^4^ M^−1^s^−1^ ^18^. The difference of approximately 1 order of magnitude between the activity of the 2 types of construct agrees with that seen between activated and not activated SOS activities in the same construct^16^. In a simple experiment, we were able to quantitate the activation of SOS activity by binding of Ras:GTP to the activator domain directly. In the experiment shown in Fig. 3B, in which mantdGDP was displaced from its complex with KRas either by GDP or by GTP in the presence of a catalytic concentration of the SOS construct. In the presence of GDP, there is a slow release of mantdGDP in a process that could be well fitted by a simple exponential equation, allowing calculation of a *k*_*cat*_ */K*_*M*_ value under these conditions of 3.2 × 10^3^ M^−1^ s^−1^. In the presence of GppNHp instead of GDP, the reaction was much faster and the initial amplitude of the reaction could not be monitored. A fit with a single exponential equation gave an apparent *k*_*cat*_ */K*_*M*_ of 4.4 × 10^4^ M^−1^ s^−1^. When a similar reaction was monitored in a stopped-flow device (Fig. 3C), sigmoidal behavior could be observed due to the allosteric activation of SOS by Ras:GppNHp formed during the reaction. We were able to fit the data obtained assuming that the Ras:GTP produced during the reaction bound to SOS and activated it to a give a *k*_*cat*_ */K*_*M*_ value of 1.03 × 10^5^ M^−1^s^−1^ for the activated form of SOS and 5.0 × 10^3^ M^−1^s^−1^ for the basal state. This suggests a degree of activation of a factor of ca. 20 when the allosteric site on SOS is occupied by Ras:GTP. The affinity of KRas:GTP to the allosteric site of SOS estimated from these experiments was given by the K_d_ value of 12.6 µM. This is in quite good agreement with a recently reported value (K_d_ = 10 µM)^19^ although not with an earlier measurement that indicated a higher affinity (K_d_ = 3.6 µM)^17^. A more detailed investigation would be needed to resolve this apparent discrepancy that might result from different mutant proteins and/or nucleotides used in the experiments.

The experiments described lead to the conclusion that the interaction of mantdGDPβMe is highly sensitive to SOS, even when the latter is not activated by occupancy of its allosteric site. The value of the limiting rate constant for mantdGDPβMe release cannot be estimated from the limited concentration range of SOS that we could use as shown in Fig. 3A, but numerical simulations of the exchange reaction suggested that it must be much larger than the 5 s^−1^ measured at 5 µM SOS concentration, since the measurements would otherwise not lead to the strictly exponential curves observed in the individual experiments contributing to Fig. 3A. Using 100 s^−1^ (i.e. a maximal acceleration by a factor of ca. 100 by SOS) for the maximal rate constant for mantdGDPβMe release, and increasing the rate constant of dissociation of SOS from KRas by the same factor to maintain thermodynamic consistency, leads to curves that are essentially exponential and in the range of rates seen, but it is possible that the rate constant is considerably higher than 100 s^−1^.

### Simulation of the kinetics of irreversible inhibition of KRas_G12C_

**Figure 4:**
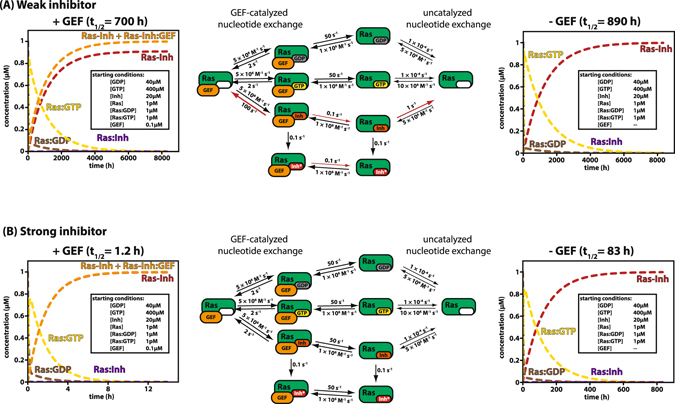
Simulation of covalent inhibition of KRas_G12C_ *in vivo*. (**A**) The kinetic parameters obtained here and in previous publications were used to simulate the covalent inhibition of KRas_G12C_ *in vivo*. Using an inhibitor with the properties of SML-8-73-1 (which is referred to as a weak inhibitor due to the low reversible affinity), the simulations led to half-lifes of covalent reaction of 890 h in the absence and 700 h in the presence of a nucleotide exchange factor. Non-covalent interactions are indicated by a colon (Rab:GDP in brown, Rab:GTP in yellow and Rab:Inhibitor in violet), covalent interactions by a hyphen (free Ras-Inhibitor in red and total concentration of Ras-Inhibitor including the fraction bound to GEF in orange). The simulation in the presence of GEF shows that a significant fraction of the covalently locked Ras-Inhibitor is bound to SOS because of the high affinity of the GEF to the Ras-Inhibitor complex. (**B**) The simulation was repeated with a (theoretical) inhibitor that retains the high affinity of binding known from GDP and GTP (and is therefore termed “strong” inhibitor). Note the dramatic decrease of half-lifes of the covalent reaction both in the absence (83 h) and especially in the presence (1.2 h) of a nucleotide exchange factor compared to the situation with the weak inhibitor (simulation files are available on request and can be opened with KinTek Explorer (http://kintekcorp.com/software/).

In order to apply the information obtained on the effects of β-phosphate group modification on the potential for such derivatives for use as covalent inhibitors of KRas_G12C_, we performed kinetic simulations on a system containing KRas, GTP, GDP and reactive GDP derivatives. The rate constants used are mainly from the literature, except those for the inhibitor or hypothetical inhibitor. For the purposes of these simulations, we have ignored the complex regulation of SOS activity^16, 17^ and used kinetic constants derived for an isolated catalytic domain^18^. The values for kinetic constants obtained for SML-8-73-1 interaction with KRas_G12C_ in the present work were used here, and the results obtained were compared with those obtained using a hypothetical GDP derivative that retains the basic (reversible) affinity and kinetics of GDP, but can react covalently with KRas. The simulations begin with KRas (1 µM) mainly in the GDP bound form and are started by virtual mixing with the covalently reacting GDP analog at 20 µM, as well as GTP and GDP at physiologically realistic concentrations (400 and 40 µM, respectively^20^). As shown in Fig. 4A, in the absence of an exchange factor, the half-life (t_1/2_) for production of the covalent adduct is ca. 890 h, which is almost certainly too slow for a significant effect in cells, even if the rate is likely to be faster at physiological temperatures rather than the 25 °C used for the constants of Fig. 4. Increasing the inactivation rate constant to 1 s^−1^ leads to a reduction of the t_1/2_ to ca. 162 hr, but this still appears too slow for the desired inhibition effect in cells.

The very slow rates of inactivation predicted from the kinetic constants measured here initially appear to be in conflict with results reported on the rate of inactivation of KRas_G12C_ by SML-8-73-1 in the presence of a 20 fold excess of GTP and GDP^21^, which was much faster than predicted here (t_1/2_ at 37° = 12 h). However, these experiments were performed in the absence of added Mg^2+^ ions, which would lead to an increase in the rate constant for GDP release from its complex with KRas. Reduction of the Mg^2+^ concentration from a physiologically realistic value to 0.1 µM leads to an acceleration of GDP release by a factor of more than 200^22^. In the experiments reported in reference^21^, the free Mg^2+^ concentration is poorly defined, but without added Mg^2+^ is likely to be very low. A combination of this with the higher temperature is probably responsible for the higher inactivation rate seen, but it should be noted that a physiologically normal Mg^2+^ concentration is important for assessing the inhibition under investigation. The effect of the Mg^2+^-concentration is experimentally addressed in the last section of our results).

We now consider a hypothetical covalent inhibitor that, in contrast to the β-phosphate modified inhibitor discussed above retains the extremely high affinity of GTP/GDP for Ras. In this case, the t_1/2_ for inactivation under the conditions described above (without GEF) is found to be ca. 83 h (Fig. 4B), i.e. approximately an order of magnitude faster than for the β-phosphate modified inhibitor considered previously. This relatively small improvement with inhibitors that bind with 10^4^ fold increased affinity can be understood on the basis of the efficient initial recognition of the weakly bound β-phosphate modified inhibitors and the relative rate constants for dissociation (1 s^−1^) and covalent reaction (0.1 s^−1^) for the weaker inhibitor, which means that approximately 1 in 10 binding events leads to covalent reaction. If *k*_*inact*_ is increased to the admittedly unlikely value of 10 s^−1^ for a β-phosphate modified inhibitor, ca. 99% of binding events will lead to covalent reaction at almost the same rate as for the more strongly bound inhibitor (t_1/2_ ≈ 100 h), but further rate acceleration does not result from even higher reactivity. Conversely, in the case of a strongly reversibly bound inhibitor, reduction of the rate of the covalent reaction from 0.1 s^−1^ by a factor of 100 has almost no effect on the rate of inactivation, since *k*_*inact*_ is still much larger than the rate constant for dissociation, meaning that practically all inhibitor molecules initially bound to the Ras mutant will undergo covalent reaction (Fig. S1).

We have so far ignored the potential influence of an exchange factor on the inactivation kinetics. It is possible, indeed likely, that it is actually more relevant to consider the inactivation kinetics in the presence of GEF activity, which in the cell means when a signal has been received that leads to activation/recruitment of an exchange factor, in particular SOS, or in KRas_G12C_ transformed cells probably even in the absence of a signal. We have therefore repeated the simulations in the presence of a GEF, using kinetic data available in the literature from our previous work on the GEF properties of Cdc25 domains^18^. We have used approximations to the measured constants, but the major uncertainty in these simulations is the effective concentration of the GEF relative to Ras. Estimates of the number of Ras molecules in the cell and the numbers of EGFR molecules lead to similar results for the 2 proteins^23^, and we have therefore performed simulations at substoichiometric, at stoichiometric and at suprastoichiometric GEF concentrations with respect to the Ras concentration (Fig. S2). However, it should be noted that the concentrations of the Ras specific GEFs SOS1 and SOS2 appear to be considerably lower^23^, so that the more realistic scenario is for the GEF to be present at lower stoichiometry. On the basis of the experimental data for the effect of GEF on the rate constant for release of a β-modified GDP reported above, we have assumed it to be accelerated by a factor of ca. 100 compared to the situation without GEF. Apart from the arguments based on experimental data presented above, a rational for this is that in structures of complexes between GTPases and GEFs in which nucleotides are bound (e.g. ref. 24), there is no interaction between the equivalent of Phe28 in Ras with the purine moiety and this is one of the major contributing factors to the enhancement of nucleotide release by GEFs. An estimate of the magnitude of the effect of loss of interaction of Phe28 with the guanine base is provided by data on the HRas_F28L_ mutant, which show that the rate constant for GDP dissociation is increased by a factor of ca. 140^25^. We have therefore chosen a rate constant of 100 s^−1^ for dissociation of the β-phosphate modified inhibitor from its complex with Ras:GEF (i.e. a factor of 100 faster than from its complex with Ras). For thermodynamic consistency, the rate constant for release of GEF from the ternary GEF:Ras:inhibitor complex is also only accelerated by a factor of 100 in comparison with the binary GEF:Ras complex, rather than the factor of more than 10^4^ for complexes with intact GDP or GDP-like structures. Fig. 4 shows the effect of 0.1 µM GEF (the KRas concentration was 1 µM). It can be seen that the rate of inactivation using the weakly bound inhibitor is enhanced under these conditions, with a half-life now of ca. 700 h (890 h without GEF). However, the rate is enhanced much more for the strongly bound inhibitor (t_1/2_ = 1.2 hours). The general explanation for the more rapid inactivation is that the originally bound GDP, and subsequently bound GTP, is released more rapidly in the presence of GEF activity, but on the negative side, the residence time of a reversibly bound inhibitor molecule on Ras is also decreased, thus decreasing the probability of covalent reaction. The latter effect is more significant for the weakly bound than the strongly bound inhibitor, since at 0.1 µM GEF the effective rate constant for release of the inhibitor will be larger than the rate constant for the covalent reaction for the weakly bound inhibitor, whereas it will still be much slower than the rate of inactivation for the strongly bound inhibitor. Increasing the GEF concentration to 1 µM leads to a further acceleration of the rate of inactivation in both cases, but again more dramatically for the tightly bound than the weakly bound inhibitor, and this trend is extended when using an excess of GEF over Ras (Fig. S2).

A major difference between the two scenarios is the fate of the GEF molecules in these simulation experiments. In the case of the strongly bound inhibitor using a concentration of GEF of 0.1 µM, there is virtually no change from the starting concentration after an initial rapid drop of ca. 0.2% in the free GEF concentration. This change is so small because of the very low affinity of any of the Ras:nucleotide complexes, including the covalent adduct, to GEF. In the case of the weakly bound inhibitor, there is a slow decrease in the free GEF concentration to a level of ca. 10^−8^ M, and this is due to the fact that with the relatively small loss of GEF affinity to Ras after the covalent reaction, much of the free GEF is sequestered at the prevailing concentrations. However, without more detailed information on the affinity of the KRas:SOS interaction when the inhibitor is bound it is difficult to make exact predictions.

### Testing the predictions from the simulations for SML-8-73-1

In order to test the predictions arising from the simulations in the case of SML-8-73-1, we have performed a number of semi-quantitative experiments using mass spectrometric analysis. Reproducing the conditions chosen for the simulations, with the exception of using a higher concentration of KRas (10 µM instead of 1 µM) to improve sensitivity, the results shown in Fig. S3A were obtained. In this experiment at 25°, almost no reaction was detected even after 52 h. This compares to a predicted half-life of ca. 1000 h from simulation under these conditions. If the K_d_ of SML-8-73-1 were indeed 0.009 µM, as reported by Xiong *et al*.^12^, the half life would be much shorter and not commensurate with the results of Fig. S3A.

To allow comparison with published work, further experiments were performed at 37°. In the first experiment, the conditions described by Hunter *et al*.^21^ were duplicated (15 µM KRasG12C, 150 µM SML-8-73-1, no added Mg^2+^ ions). The actual free Mg^2+^ concentration in this experiment was ca. 20 µM (arising from the KRas stock solution), as it probably was in the Hunter *et al*.^21^ work. As shown in Fig. S3B, 50% reaction occurred in 1–2 h, compared with the half-life of 0.7 h. reported by Hunter *et al*. This difference is probably not significant and could be due to the differences in the poorly defined Mg^2+^ concentration. Repetition of this experiment with the addition of 1.5 mM GTP and 1.5 mM GDP as used by Hunter *et al*. (note that these values are actually much higher than physiological concentrations) led to a much slower reaction, with a half-life in the region of 40 h (Fig. S3C), compared with 12 h in the published work. Addition of 1 mM Mg^2+^ in the absence of excess GTP/GDP also leads to a slower rate of reaction, with the half-life now being ca. 8 h (Fig. S3D). Addition of both 1.5 mM each of GTP and GTP and 3 mM Mg^2+^ led to a much slower reaction (a few percent after 51 h; Fig. S3E), but at the elevated temperature, protein denaturation (as evidenced by precipitation) made it impossible to follow the time course to estimate the half-life.

In an experiment designed to achieve a maximal rate of inactivation, no excess GTP/GDP was added, and the Mg^2+^ concentration was depleted even further using EDTA. In this case, there was very rapid generation of the covalent adduct, so that the first time of measurement (designated as time zero in the figure, but in practice after ca. 10 min), the reaction was practically complete (Fig. S3F).

## Conclusions

The work undertaken here had the aim of determining the effect of modification of the β-phosphate group of GDP on the affinity and kinetics of interaction with KRas. The motivation for this was to test the possible applicability of GDP derivatives equipped with a chemically reactive group at this position for the targeted covalent modification of oncogenic KRas mutants, in particular KRas_G12C_. We have used the smallest easily available group, a methyl group, for this purpose, since it will have similar consequences for the initial, non-covalent binding to that of the already reported GDP modification used to interact covalently with KRas_G12C_^11^. As shown above, modification of the phosphate group by esterification leads to a dramatic loss of affinity to KRas when compared with GDP. The major contribution to this loss of affinity is without doubt the loss of one of the 2 negative charges on the β-phosphate group. This group is involved in a large number of interactions, one of the most important of which is that with Lys16 of the so-called P-loop^15^. Additionally, there is an interaction of another β-phosphate oxygen with a magnesium ion. The loss of the negative charge is likely to weaken one or both of these interactions significantly. It is highly probable that the Mg^2+^ affinity to the KRas:nucleotide complex is reduced considerably from its known value to Ras:GDP complexes (K_d_ = 2.8 µM^22^), and even possible that at physiological Mg^2+^ concentrations the metal ion would not be bound. In addition to these important and essential interactions (for high affinity), there are many backbone (amide NH group) interactions with the β-phosphate^15^, a number of which could be eliminated or weakened by β-phosphate modification. Examination of SML-8-73-1 using similar methods led to the conclusion that the affinity of this derivative to KRas or KRas_G12C_ was similar to that of GDPβMe.

To examine the potential effect of such modifications on the efficacy of KRas_G12C_ inhibition by β-modified GDP derivatives, we performed kinetic simulations with known rate constants for elementary steps in the mechanism. Using kinetic constants determined here and elsewhere, we show that the dramatically reduced affinity of such derivatives has a marked negative effect on the irreversible inhibition of the mutant compared to a hypothetical situation in which the covalent inhibitor has the same or similar affinity and kinetics of interaction as GDP/GTP in a situation in which we consider only the mutant, the GDP-based inhibitor and GTP/GDP (at a physiologically relevant concentrations) are present. Thus, although the inhibitor can compete well with GDP/GTP in initial binding to KRas, the rapid rate of release (0.8 s^−1^) means that unless the covalent reaction occurs with a rate constant that is considerably larger than this value, many binding and dissociation events will occur before covalent reaction can take place. The efficiency of any inhibitor of the type represented by SML-8-73-1 will in fact be highly dependent on the rate constant of the covalent reaction in comparison with the rate constant of release. Thus, using the measured value of 0.1 s^−1^, this means that a number of association and dissociation events will occur on average before the covalent reaction can take place, and the frequency of these events is reduced dramatically by competition with GTP/GDP. The situation would be improved considerably if k_inact_ were much larger than the dissociation rate constant (e.g. if k_inact_ was 10 s^−1^, most binding events would lead to covalent reaction).

The situation would be quite different with a covalent inhibitor that bound with a reversible affinity of a similar order of magnitude to that of GDP/GTP. Here, while competition with the initial binding to Ras will not be different to that seen with SML-8-73-1, the long life-time of the bound state (dictated by the low dissociation rate constant of 10^−4^ s^−1^) will mean that essentially all binding events will lead to covalent interaction. This will even be true if the rate of covalent modification is considerably slower than the 0.1 s^−1^ assumed for the simulations, so that even with a rate constant of 0.001 s^−1^, most binding events (ca. 90%) will lead to covalent reaction (Fig. S1). This is potentially a major advantage of such hypothetical inhibitors, since a high reactivity of the electrophilic group used will inevitably lead to significant side reactions in the cellular context and to inactivation by thiol containing small molecules such as glutathione.

In the presence of GEF activity, the situation changes dramatically, with the general effect of more rapid inactivation of Ras due to a higher rate of GDP/GTP dissociation. The effect is much more dramatic for strongly binding inhibitors than for the weakly bound β-phosphate modified inhibitors, due to the fact that considerable acceleration of the rate of GDP/GTP release can be tolerated without the probability of the covalent reaction occurring being reduced. It seems highly like that GEF activity will be high in KRas_G12C_ transformed cells due to the recruitment of SOS to the plasma membrane via interaction of its allosteric site with activated KRas_G12C_, thus allowing activation of further Ras molecules. There is indeed strong evidence that this is a significant effect, since SOS induced cross-activation of wild type Ras isoforms by oncogenic forms has been demonstrated^26^.

The design of covalent inhibitors that retain the crucial features of GDP or GTP, in particular the unmodified phosphate groups and the guanine base, but also bear a chemically reactive group, is not a trivial task. Thus, although specific interactions with the ribose moiety of GDP/GTP do not appear to be important for high affinity, the removal of constraints on the overall sugar structure in acyclovir triphosphate described above leads to a large drop in affinity, all of which arises from the increased dissociation rate. This suggests that retention of the basic ring structure of the ribose moiety, or an alternative structure that positions the phosphates and the guanine base in a similar conformation, will be required for high affinity.

Possible positions for attachment of an electrophilic group would be the 2′- and 3′ positions, but these are probably too remote from amino acids 12 or 13 in the Ras structure to position an electrophilic group properly. Similar considerations also apply to already described derivatives that have a reactive group attached to the exocyclic N2 position of the guanine base^27^. Sterically more interesting are the 5′-carbon, while still retaining the phosphorylated hydroxyl group, or the 4′ carbon. Both modifications are synthetically challenging, but not impossible to achieve.

## Materials and Methods

### Nucleotides

All nucleotide analogs were obtained from Jena Bioscience GmbH (Jena, Germany).

### Protein expression and purification

KRas_WT_ and KRas_1–169_ G12C C51S C80L C118S were cloned into a modified pET19 expression vector containing a His6-tag and a PreScission cleavage site. The proteins were expressed after induction with 0.5 mM IPTG overnight at 20 °C. Subsequently they were purified by Ni^2+^-IMAC, cleavage of the His-tag, reverse Ni^2+^-IMAC and size exclusion chromatography in a final buffer containing 20 mM Hepes pH 7.5, 50 mM NaCl, 1 mM TCEP, 10 µM GDP, 2 mM MgCl_2_. Nucleotide-free protein was prepared by buffer exchange to 20 mM HEPES pH 7.5, 100 mM NaCl, 160 mM (NH_4_)_2_SO_4_, 2 mM DTE, addition of 5 mM EDTA, 10-fold excess GppCH_2_p and 2.5 units alkaline phosphatase, and incubation overnight at 4 °C. GDP degredation was monitored by HPLC (column Prontosil 120-5-C18 5 µm; buffer 50 mM KP_i_ pH 6.6, 10 mM tetrabutylammonium bromide, 16% acetonitril). Degradation of GppCH_2_p was performed by addition of 1 unit phosphodiesterase per mg protein and 500 µM ZnCl_2_ at room temperature and monitored by HPLC as above. Finally, the protein was purified by size exclusion chromatography (buffer: 20 mM Hepes pH 7.5, 160 mM (NH_4_)_2_SO_4_, 1 mM TCEP).

SOS_564–1049_ was cloned into a pET28 expression vector and purified as above, but without cleavage of the His-tag. Final purification by size exclusion chromatography was carried out in 20 mM Hepes pH 7.5, 50 mM NaCl, 1 mM TCEP.

### Stopped-flow experiments

All stopped-flow experiments were performed in a SX-20 stopped-flow apparatus (Applied Photophysics) at 25 °C in a buffer consisting of 20 mM Hepes pH 7.5, 100 mM NaCl, 1 mM TCEP, 2 mM MgCl_2_ (excitation with 360 nm LED, emission detection with a 420 nm cut-off filter).

For competition experiments, binding of 1 µM nucleotide free wild-type KRas (referred to as KRas_WT_ in the following) or KRas_1–169_ G12C C51S C80L C118S (referred to as KRas_G12C_) to 1 µM mantdGDP or 1 µM mantdGDPβMe in the absence of or the presence of 0.5 µM, 1 µM and 3 µM of competing nucleotides (GDP, GDPβMe, acyclovir or SML-8-73-1) was assessed and the resulting progress curves were globally fit using KinTek Explorer (the corresponding files are available as a supplement and can be opened with KinTek Explorer^28^ (http://kintekcorp.com/software/).

SOS catalyzed nucleotide exchange on 200 nM KRas:mantdGDP or KRas:mantdGDPβMe was characterized by exchange with 50 µM GDP in the presence of different concentrations of SOS, the resulting progress curves were fit with a single exponential equation and observed rate constants were plotted against the SOS concentration to obtain *k*_*cat*_ */K*_*M*_ from the slope of the linear fit (Fig. 3A).

Allosteric activation of SOS was tested by mixing 10 µM KRas:mantdGDP with 1 µM SOS/1 mM GppNHp (Fig. 3C).

### Slow kinetics

Slow kinetics of nucleotide exchange in the presence of catalytic amounts of SOS were measured in a fluoromax-3 spectrofluorometer (excitation at 360 nm, emission 440 nm) at 25 °C in 20 mM Hepes pH 7.5, 100 mM NaCl, 1 mM TCEP, 2 mM MgCl_2_. 10 µM KRas:mantdGDP was incubated in the presence of 0.5 µM SOS and the reaction was started by addition of 1 mM GDP or 1 mM GppNHp.

### Electrospray ionization mass spectrometry (ESI-MS)

The covalent reaction of SML-8-73-1 with KRas_G12C_ was monitored by mass spectrometry. 200 µM SML-8-73-1 was added to the nucleotide-free protein (50 µM), 5 mM dithioerythritol (DTE) was added after ~20 s and the extent of modification monitored by ESI-MS.

For the measurements described in Fig. S3, KRas_G12C_ and SML 8-73-1 were incubated in 20 mM HEPES pH 7.5 and 50 mM NaCl. The conditions are as mentioned in the figure. For each time point, the reaction was stopped by the addition of DTT (to 5 mM final concentration) and acetonitrile (to 25%).

### Kinetic simulations

Kinetic simulations were performed using KinTek Explorer. The corresponding files will be made available by the corresponding authors upon request and can be opened with KinTek Explorer^28^ (http://kintekcorp.com/software/).

### Data availability

Data will be made available upon request to the corresponding authors.

## Electronic supplementary material

Supplementary material

## References

[1] Prior IA, Lewis PD, Mattos C (2012). A comprehensive survey of Ras mutations in cancer. Cancer Res.

[2] Cox AD, Fesik SW, Kimmelman AC, Luo J, Der CJ (2014). Drugging the undruggable RAS: Mission possible?. Nature reviews. Drug discovery.

[3] John J (1990). Kinetics of interaction of nucleotides with nucleotide-free H-ras p21. Biochemistry-Us.

[4] Goody RS, Frech M, Wittinghofer A (1991). Affinity of Guanine-Nucleotide Binding-Proteins for Their Ligands - Facts and Artifacts. Trends Biochem Sci.

[5] Berndt N, Hamilton AD, Sebti SM (2011). Targeting protein prenylation for cancer therapy. Nature reviews. Cancer.

[6] Whyte DB (1997). K- and N-Ras are geranylgeranylated in cells treated with farnesyl protein transferase inhibitors. J Biol Chem.

[7] Zimmermann G (2013). Small molecule inhibition of the KRAS-PDEdelta interaction impairs oncogenic KRAS signalling. Nature.

[8] Spiegel J (2014). Direct targeting of Rab-GTPase-effector interactions. Angew Chem Int Ed Engl.

[9] Ostrem, J. M., Peters, U., Sos, M. L., Wells, J. A. & Shokat, K. M. K-Ras(G12C) inhibitors allosterically control GTP affinity and effector interactions. *Nature* **503**, 548-+, doi:10.1038/nature12796 (2013). 10.1038/nature12796 PMC4274051 24256730

[10] Patricelli MP (2016). Selective Inhibition of Oncogenic KRAS Output with Small Molecules Targeting the Inactive State. Cancer discovery.

[11] Lim SM (2014). Therapeutic Targeting of Oncogenic K-Ras by a Covalent Catalytic Site Inhibitor. Angew Chem Int Edit.

[12] Xiong Y (2017). Covalent Guanosine Mimetic Inhibitors of G12C KRAS. ACS medicinal chemistry letters.

[13] Nowak E, Goody RS (1988). Kinetics of Adenosine 5′-Triphosphate and Adenosine 5′-Diphosphate Interaction with G-Actin. Biochemistry-Us.

[14] Rensland H (1995). Substrate and product structural requirements for binding of nucleotides to H-ras p21: the mechanism of discrimination between guanosine and adenosine nucleotides. Biochemistry-Us.

[15] Pai EF (1990). Refined Crystal-Structure of the Triphosphate Conformation of H-Ras P21 at 1.35 a Resolution - Implications for the Mechanism of Gtp Hydrolysis. Embo J.

[16] Margarit SM (2003). Structural evidence for feedback activation by Ras.GTP of the Ras-specific nucleotide exchange factor SOS. Cell.

[17] Sondermann H (2004). Structural analysis of autoinhibition in the Ras activator Son of sevenless. Cell.

[18] Guo Z, Ahmadian MR, Goody RS (2005). Guanine nucleotide exchange factors operate by a simple allosteric competitive mechanism. Biochemistry-Us.

[19] Vo U (2016). Monitoring Ras Interactions with the Nucleotide Exchange Factor Son of Sevenless (Sos) Using Site-specific NMR Reporter Signals and Intrinsic Fluorescence. J Biol Chem.

[20] Traut TW (1994). Physiological Concentrations of Purines and Pyrimidines. Mol Cell Biochem.

[21] Hunter JC (2014). *In situ* selectivity profiling and crystal structure of SML-8-73-1, an active site inhibitor of oncogenic K-Ras G12C. Proc Natl Acad Sci USA.

[22] John J (1993). Kinetic and Structural-Analysis of the Mg2+-Binding Site of the Guanine Nucleotide-Binding Protein P21(H-Ras). J Biol Chem.

[23] Shi T (2016). Conservation of protein abundance patterns reveals the regulatory architecture of the EGFR-MAPK pathway. Sci Signal.

[24] Guo Z, Hou X, Goody RS, Itzen A (2013). Intermediates in the guanine nucleotide exchange reaction of Rab8 protein catalyzed by guanine nucleotide exchange factors Rabin8 and GRAB. J Biol Chem.

[25] Reinstein J, Schlichting I, Frech M, Goody RS, Wittinghofer A (1991). P21 with a Phenylalanine 28-] Leucine Mutation Reacts Normally with the Gtpase Activating Protein Gap but Nevertheless Has Transforming Properties. J Biol Chem.

[26] Jeng, H. H., Taylor, L. J. & Bar-Sagi, D. Sos-mediated cross-activation of wild-type Ras by oncogenic Ras is essential for tumorigenesis. *Nature communications* **3**, 1168 10.1038/ncomms2173 PMC3640996 23132018

[27] Wiegandt D (2015). Locking GTPases covalently in their functional states. Nature communications.

[28] Johnson KA, Simpson ZB, Blom T (2009). Global kinetic explorer: a new computer program for dynamic simulation and fitting of kinetic data. Anal Biochem.

