# Supplementary material for: Nucleotide based covalent inhibitors of KRas can only be efficient in vivo if they bind reversibly with GTP-like affinity
Source: Sci Rep. 2017 Jun 16;7:3687. doi: 10.1038/s41598-017-03973-6 (PMC5473928; doi:10.1038/s41598-017-03973-6)
Supplement: Supplementary file 1 — Supplementary material [file 41598_2017_3973_MOESM1_ESM.pdf]

Nucleotide based covalent inhibitors of KRas can only be efficient *in vivo* if they bind reversibly with GTP-like affinity

Supplementary material

Matthias P. Müller[1,2], Sadasivam Jeganathan[1], Angelika Heidrich[1], Jeremy Campos[1] and Roger S. Goody[1]

[1]Department of Structural Biochemistry, Max Planck Institute of Molecular Physiology, Otto-Hahn-Straße 11, 44227 Dortmund, Germany
and
[2]Faculty of Chemistry and Chemical Biology, Dortmund University of Technology, Otto-Hahn-Straße 4a, D-44227 Dortmund, Germany

Correspondence:
Matthias P. Müller (matthias3.mueller@tu-dortmund.de)
Roger S. Goody (goody@mpi-dortmund.mpg.de)

Supplementary Figure 1

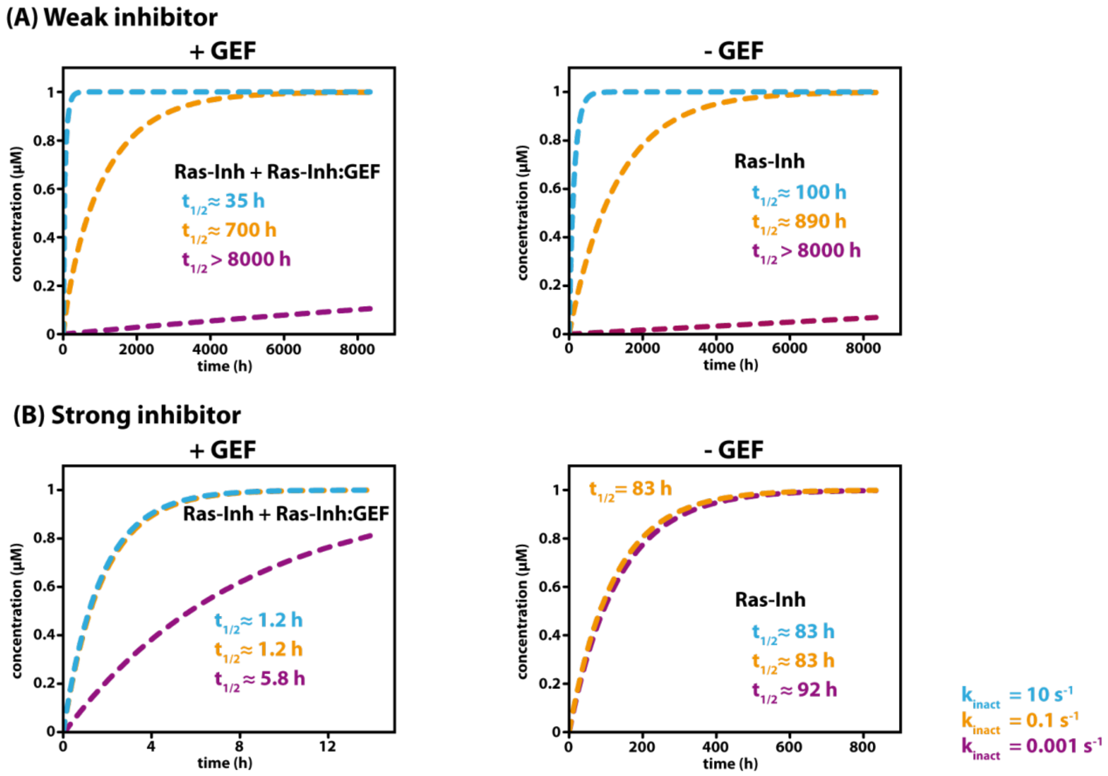

Supplementary Figure 1: The effect of k_{inact}. Simulations were repeated as shown in the main manuscript, but varying the rate constant of covalent reaction (k_{inact}) of the inhibitor with $KRas_{G12C}$. Whereas changes in the rate of covalent reaction have a strong impact on the half-life of the reaction for the weakly bound inhibitor **(A)**, the effect is less dramatic for the strongly bound inhibitor **(B)** and physiologically reasonable life-times can still be achieved with inhibitors that have a low reactivity and are therefore presumably more stable and selective *in vivo* regarding side reactions and inactivation of the inhibitor by e.g. glutathione.

Supplementary Figure 2

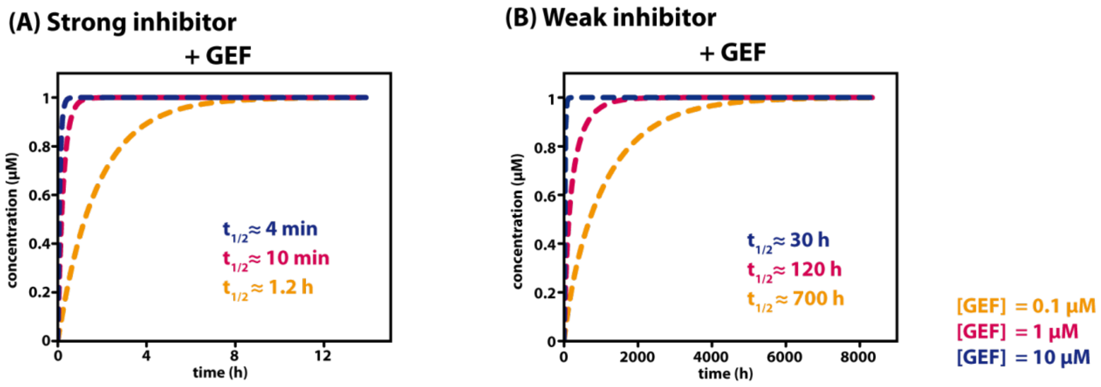

Supplementary Figure 2: The effect of the concentration of GEF. Simulations were made for the rate of covalent reaction of an inhibitor with $KRas_{G12C}$ using a strongly **(A)** or a weakly **(B)** binding inhibitor (k_{inact} was set to 0.1 s^{-1} in both cases).

Supplementary Figure 3

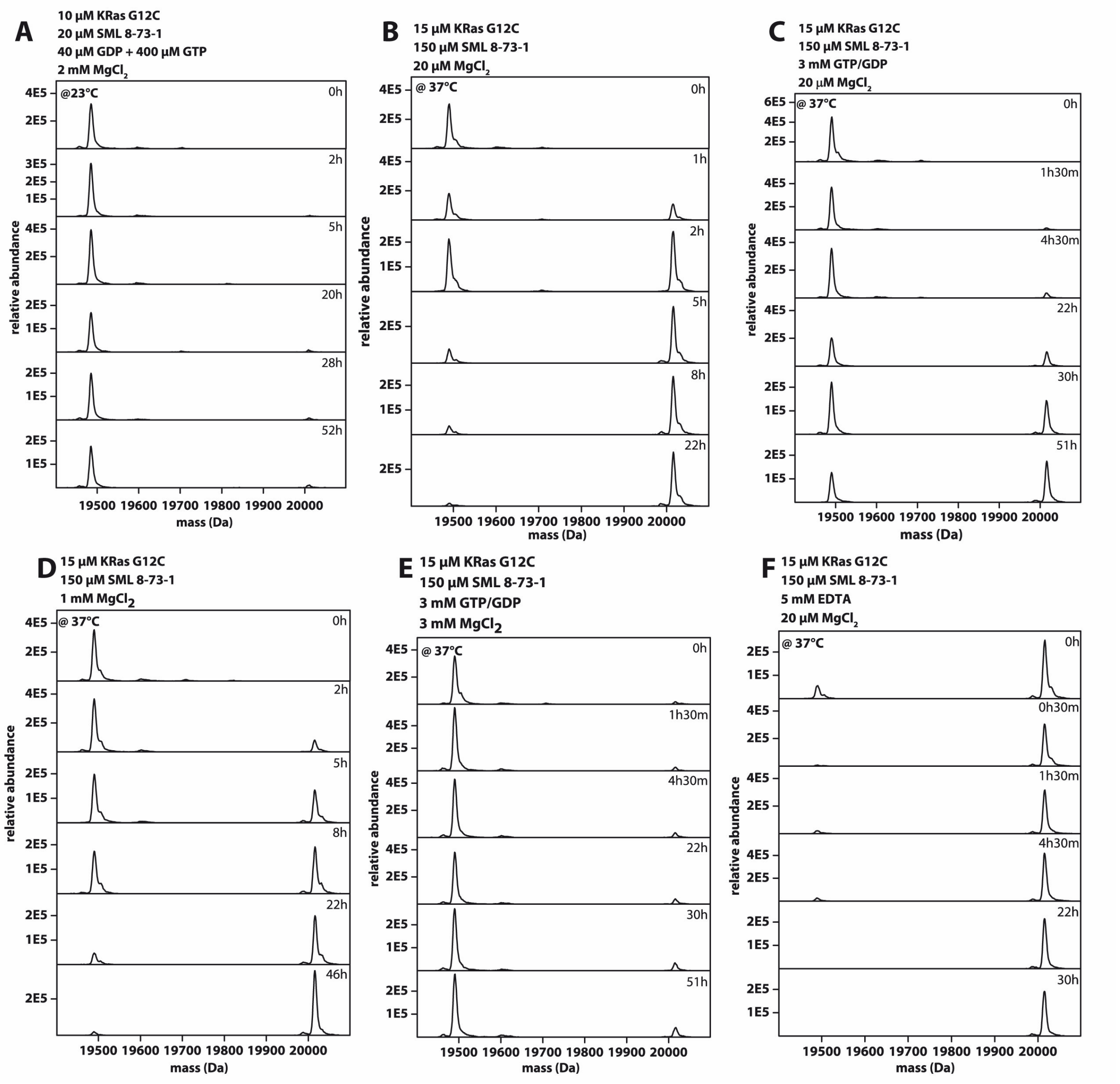

Supplementary Figure 2: Electrospray ionization mass spectrometric analysis (ESI-MS) of the covalent modification of KRasG12C by SML-8-73-1 under different conditions. The unmodified protein is seen at a mass of just under 19,500, and after modification at just over 20,000. The conditions are given for each reaction.
